# The Comparison of Microwave Thawing and Ultra-High-Pressure Thawing on the Quality Characteristics of Frozen Mango

**DOI:** 10.3390/foods11071048

**Published:** 2022-04-06

**Authors:** Yu Peng, Jinhong Zhao, Xin Wen, Yuanying Ni

**Affiliations:** 1 College of Food Science and Nutritional Engineering, China Agricultural University, No. 17 Qinghua East Road, Beijing 100083, China; yu1.peng@outlook.com (Y.P.); nyy@cau.edu.cn (Y.N.); 2 Beijing Academy of Food Sciences, Beijing 100068, China; oriental123@126.com

**Keywords:** food loss, microwave thawing, ultra-high-pressure thawing, mangoes, quality attributes

## Abstract

As one of the popular tropical fruits, mango has a relatively short shelf life due to its perishability. Therefore, post-harvest losses are always a topic of concern. Currently, freezing is a common approach to extending mango shelf life. In relation, it is also critical to select a proper thawing process to maintain its original quality attributes. In this study, microwave thawing, and ultra-high-pressure thawing were investigated, and traditional thawing methods (air thawing and water thawing) were compared as references. The thawing time, quality attributes, and sensory scores of frozen mangoes were evaluated. Compared to traditional methods, innovative thawing methods can extensively shorten thawing time. These things considered, the thawing time was further decreased with the increase in microwave power. Additionally, microwave thawing enhanced the quality of mangoes in terms of less color change and drip loss and reduced loss of firmness and vitamin C content. Microwave thawing at 300 W is recommended as the best condition for thawing mangoes, with the highest sensory score. Current work provides more data and information for selecting suitable thawing methods and optimum conditions for frozen mango to minimize losses.

## 1. Introduction

The food crisis generated by the scarcity of food supplies has become an important issue to feed the global population, whereas food loss has been increasingly developed over the past decades to contrast the global food crisis [1,2]. Mango (*Mangifera indica* L.) is one of the most economically important fruits worldwide, especially in the Asia area [3]. It is popularly referred to as the “King of Fruits” with attractive flavors and excellent taste [4,5]. Moreover, the health-beneficial compounds in mango, such as vitamin C, carotenoids and phenolic compounds, provide a good source of antioxidants, which can potentially reduce the risk of some diseases such as diabetes [6,7]. However, the perishability of mango can cause the magnitude of post-harvest losses during storage, transportation, and market practice, accounting for a huge economic drain on the global market [8]. The freezing process can be a potential option to increase the environmental and economic sustainability of mango production.

Freezing has long been established as one of the most effective ways of preserving food, which is important for quality maintenance and shelf-life extension [9]. Apart from the preservation aspects, the freezing process allows the transportation of regional and seasonal products, such as tropical fruits to remote markets, and their products to be consumed throughout the whole year [10]. Generally, the freezing process involves three operating units: actual freezing, frozen storage, and thawing, all of which were found to influence the quality attributes of frozen food products [11,12]. The thawing process is a reverse process of freezing and an essential step before any subsequent consumption or cooking [12]. Mango may undertake quality loss, such as texture destruction, color degradation and flavor changes if an improper thawing process is performed [13]. Mango suffers a decrease in quality attributes that may lead to food waste, eventually in the retail sale or during consumption. Therefore, employing suitable thawing technology and appropriate conditions is of great importance in maintaining the mango product’s quality attributes.

Traditional thawing methods, such as air thawing and water thawing, are broadly applied in industrial processing due to their low capital investments and operation simplicity [14]. However, these methods possess severe drawbacks, such as a slow thawing process, microbial reproduction, and other irreversible quality degradations [15,16]. To mitigate negative effects and minimize the losses, novel thawing methods are considered to be alternative options. Novel thawing methods generally can be grouped into thermal thawing and non-thermal thawing. Microwave thawing is a promising thermal thawing method. In principle, microwave heating is the conversion transfer of electromagnetic energy to thermal energy through the direct interaction of the incident radiation and the applied material [17]. The transfer of energy does not depend on the diffusion of heat from the surfaces, which brings the possibility to achieve a rapid thawing process of frozen food [18]. Microwave thawing offers many advantages with regard to short processing times, energy savings, improved nutritional qualities, and acceptability in some foods such as meat products and frozen fruits [19,20]. Ultra-high-pressure thawing is an innovative non-thermal thawing method with great potential for the food industry. The principle of the pressure-assisted thawing process is the decrease in the melting point. According to the phase diagram of water, the melting temperature of water decreases with an increase in pressure, down to −21 °C at 210 MPa [21]. The decreased melting point can increase the temperature gap between the thawing medium and frozen samples, and therefore, increase the thawing rate [22]. Moreover the depression of the melting point, the phase transition processes can be accelerated by a reduced enthalpy of crystallization under high pressure [23]. Therefore, the high-pressure thawing method can offer rapid thawing applications. Moreover, previous research showed that without the application of heat, high-pressure-assisted thawing can largely preserve fresh color and retain fresh flavor much more than traditional thermal processing [10,24]. Thus far, high-pressure thawing has mainly been focused on meat and seafood products, but limited studies were reported on frozen fruits and vegetables [25,26].

To date, the comparison between microwave thawing and ultra-high-pressure thawing, as the representative of thermal and non-thermal thawing methods on the quality characteristics of frozen mango has not been investigated. Therefore, to provide more data and information for selecting suitable thawing methods and optimum conditions for thawing mango, the main objective of the current work was to compare the effects of microwave thawing and ultra-high-pressure thawing on the thawing time and quality attributes of frozen mangoes. The traditional air thawing and water thawing were performed as the references.

## 2. Materials and Methods

### 2.1. Materials

Fresh ripened mangoes (Tai-Nong No. 1) were well-selected and obtained directly from a local market in Beijing, China. The physicochemical properties of fresh mangoes are presented in Table 1. The chemicals used in this study were analytical grade and obtained from Sigma Aldrich (Darmstadt, Germany). The water used throughout the study was purified by a Milli-Q Lab Water System (Milli-Q IQ 7000 Ultrapure Lab Water System, Merck KGaA, Darmstadt, Germany) unless stated otherwise.

### 2.2. Methods

#### 2.2.1. Frozen Mango Preparation

**Figure 1:**
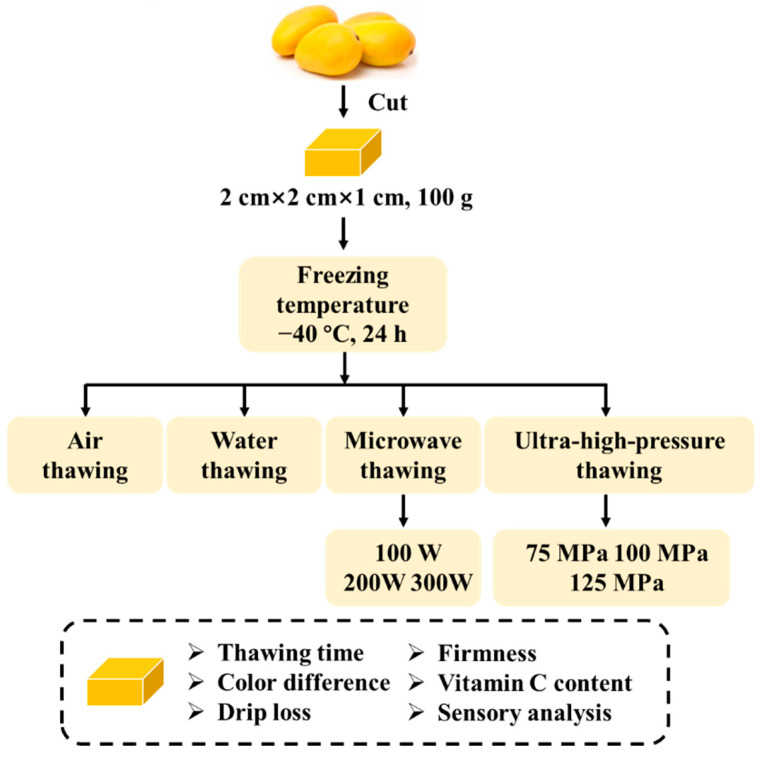
The flow chart of the experimental design.

The experimental design of this study can be found in Figure 1. The fresh mangoes were carefully picked for homogeneous properties regarding color, size and shape. They were peeled, deseeded, and cut into cuboid shapes (2 cm × 2 cm × 1 cm) manually. The cut mango was then evenly divided and placed in food-grade transparent polyethylene bags, which are applicable for microwave and ultra-high-pressure thawing processes. All the bags were vacuum-sealed subsequently and transferred to an ultra-low temperature freezer for quick freezing (DW-HL388, Zhongke Meiling Co., Ltd., Hefei, China). The weight of each bag was around 100 g, and 3 to 5 bags of mango were prepared for each thawing condition for one repetition. The freezing temperature was set to −40 ± 2 °C, and the freezing time was controlled at 24 h.

#### 2.2.2. Thawing Process

Four different thawing methods were carried out and compared as follows:

Microwave thawing (MT) was performed using a microwave oven (721NH1-PW, Midea Group Co., Ltd., Beijing, China). The frozen mangoes were thawed with microwave power at 100 W, 200 W, and 300 W, respectively. The frozen samples were consistently located in the same spot inside the microwave and heated intermittently. Overheating (run-away heating) phenomenon was observed when the microwave power exceeded 300 W.

Ultra-high-pressure thawing (UHPT) was performed in a hydrostatic pressurization unit (HHP-700; Baotou Kefa Co., Ltd., Baotou, China). The pressure transmitting medium was water, while the temperature was set at 20 ± 1 °C. The pressure 75 MPa, 100 MPa, and 125 MPa were selected to treat the frozen mangoes, respectively. The increased rate of pressure was approximately 133 MPa/min. After the thawing process, the pressure release was immediate. The pressure increase and release time were included in this study when reporting the treatment time.

Air thawing (AT) and water thawing (WT) were performed as the references, by which the frozen mangoes were placed under room temperature (20 ± 1 °C) in air and a water bath with the water temperature at 20 ± 1 °C, respectively.

The thawing process was considered complete when the center temperature of the mango sample reached 0 °C, and the thawing time was recorded. The center temperature was measured by a high-accuracy temperature sensor (FOTS-DINA-2050, Beijing, China), with which the diameter of the probe was 2 mm. All the thawing conditions were performed at least in triplicate for further analysis.

#### 2.2.3. Color Measurement

The color of fresh and thawed mango samples was examined with a chromaticity instrument (HunterLab ColorQuest XE, Hunter Associates Laboratory, Inc., Reston, VA, USA) at room temperature. Before the measurement, the equipment was calibrated by a white standard board firstly, after which the measurement results were recorded. The L* (lightness), a* (redness-greenness), and b* (yellowness-blueness) parameters of the CIELAB colorimetric system were recorded and reported. Meanwhile, the total color difference (Δ*E*) was selected to determine the effect of different thawing methods on the color change of mango samples. The color difference value was calculated according to Equation (1):

(1) $$\Delta E=\sqrt{{\left(\Delta L*\right)}^{2}+{\left(\Delta a*\right)}^{2}+{\left(\Delta b*\right)}^{2}}$$

#### 2.2.4. Firmness Measurement

The firmness of fresh and thawed mango samples was determined by a texture analyzer (TMS-Pro, Food Technology Corporation, Sterling, VA, USA) equipped with a P/38-mm diameter cylinder probe. The test mode was set as a compression test, while the mango samples were compressed twice to 30% of their initial height. The test speed was 20 mm/min, and the initial force was 0.5 N. A force–time curve was recorded and reported. The peak force of the first compression cycle was expressed as the firmness of each sample. All the samples were measured 6 times.

#### 2.2.5. Drip Loss Measurement

After the thawing process, the surface of the mango sample was well-cleaned to remove the exuded water properly. The weight of the sample was recorded before thawing (M_1_) and after thawing (M_2_). The value of drip loss was calculated based on Equation (2).

Drip Loss (%) = (M_1_ − M_2_)/M_1_ × 100% (2)

#### 2.2.6. Vitamin C Content Measurement

The 2,6-dichlorophenol-indophenol titration method was used to measure the vitamin C content of mango samples with slight changes [27]. The mg of ascorbic acid per 100 g of measured samples was reported as the values of vitamin C content.

#### 2.2.7. Sensory Analysis

The sensory analysis was designed to evaluate the overall acceptability of fresh mango and all the thawed mangoes. The panel consisted of 10 trained assessors between the ages of 20–30, with normal olfactory and gustatory at the time of the analysis. All the assessors were given sufficient information only to conduct the evaluation, while all the persons who were directly involved in preparing the samples were excluded from the panel.

In the session, 3–5 mango cuboid samples were evaluated. A traditional nine-point hedonic scale was used for the sensory evaluation, while the assessors were asked to rate the overall acceptance of the fresh mango and thawed mangoes on a scale from 1 = dislike extremely and 9 = like extremely. The sample offers were randomly arranged to minimize central tendency errors [28].

#### 2.2.8. Statistical Analysis

The thawed mango samples were prepared in triplicate for each thawing method and thawing parameter, and all the experiments were performed in triplicate at least. Data were expressed as mean ± standard deviation. SPSS 17.0 software (SPSS, Chicago, IL, USA) was used to perform the ANOVA and Duncan’s multiple range tests at a significance level of *p* < 0.05.

## 3. Results

The quality characteristics of fruits and their products are generally affected by four aspects: color (appearance), flavor (taste), texture, and nutritional value [10,29]. In this study, different thawing methods were performed on frozen mangos, while the thawing time and the quality characteristics of the thawed mangos were compared and discussed.

### 3.1. Thawing Time

**Figure 2:**
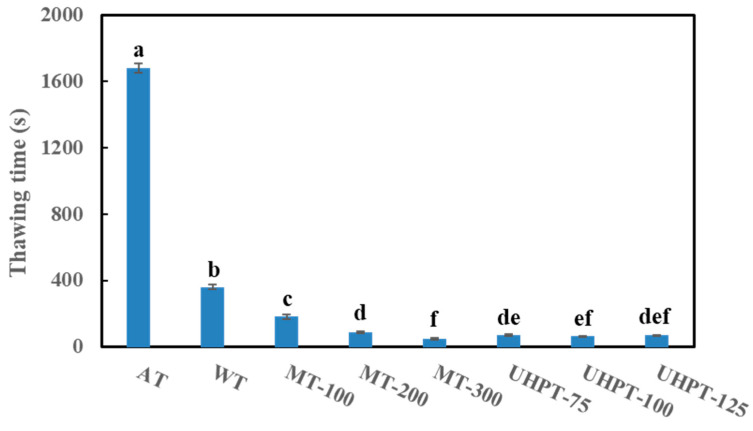
The thawing time of frozen mangoes that were affected by different thawing conditions. The different lowercase letters mean the significant difference among thawing conditions (*p* < 0.05).

The thawing time of frozen mango greatly depended on the applied thawing method and thawing parameter (Figure 2). Air thawing at room temperature took around 28 min, while the thawing time was reduced to around 6 min when water thawing was used. This was expected because the air had lower heat conductivity and specific heat than water. Similar results were also found on frozen red radish [30] and frozen strawberries [25]. When microwave thawing was performed with 100 W, the thawing time was approximately 3 min. The frozen mango samples had a much faster thawing process when the microwave power increased, achieving the shortest thawing time when 300 W was applied (48.8 s). The thawing process was also accelerated by selecting ultra-high-pressure thawing. The thawing time was 72.9 s, 64.5 s, and 69.5 s when the assisted pressure was 75 MPa, 100 MPa, and 125 MPa, respectively.

The traditional thawing methods were much more time-consuming than the innovative thawing methods, which might be caused by the heating pattern and the temperature gap between the thawing medium and frozen samples. As time went on, the heat transfer of air thawing and water thawing was from the exterior to the interior [16]. Moreover, for microwave thawing, microwave heating consists of the interaction between an electromagnetic field and molecules or particles with a non-zero charge distribution. This interaction with microwaves is mainly caused by the dipolar nature of water. The microwaves absorbed by food can excite the water molecules, and therefore generate heat [18]. Consequently, the thawing process could be accelerated by the produced heat within the food materials from microwave radiation [31]. As for ultra-high-pressure thawing, the process generally involves compression heating, pressure-dependent temperature change, phase transition and heat transfer [32]. The freezing point reduction under high pressure significantly expands the temperature difference between the frozen sample and the environment and thus increases the driving force and the thawing rate effectively.

Generally, a short thawing time is necessary to speed up frozen food processing operations. For frozen food, the enzymes and microorganisms which are de-activated by the low temperature during the freezing process and frozen storage may activate again, multiply, and stimulate spoilage if the thawing time is too long [14]. Therefore, among all the thawing methods and parameters, 300 W microwave thawing was the most efficient and achieved the quickest thawing process (48.8 s), since, for the ultra-high-pressure thawing process, the pressure increase time was also included when recording the thawing time.

### 3.2. Color Changes

The appearance in terms of fruits’ color change usually determines if a related product is acceptable or not; thus, the color change becomes one of the most critical quality attributes [33]. The color changes (Δ*E*) in mangoes after four different thawing methods are shown in Table 1. It can be found that microwave thawing and ultra-high-pressure thawing at 75 MPa and 100 MPa had significantly lower Δ*E* values of thawed mangoes than air and water thawing, which implied that microwave thawing and ultra-high-pressure thawing (75 MPa and 100 MPa) were able to maintain the samples surface color better. The hypothesis was formed that the fast-thawing rate of microwave and ultra-high-pressure thawing could reduce mechanical damage to the cell membranes and therefore, result in fewer color changes after thawing. However, when pressure achieved 125 MPa, thawed samples obtained the highest Δ*E* (12.67) values, which indicated that high-pressure processing at a higher pressure caused severe color losses to mangoes.

Table 2 shows the L*, a*, and b* values of all the thawed samples, while L* describes the lightness (L* = 100 indicates white, L* = 0 indicates black) in the color space. After the thawing process, the L* value of thawed mango decreased with the increase in process pressure, and 125 MPa-pressure-thawed mangoes presented the lowest L* value among all the thawing methods and conditions. However, the L* value of microwave-thawed samples was higher than that of other thawed samples. Compared with fresh mangoes, the reduction in the L* value of air thawing, water thawing, and ultra-high-pressure thawing (100 MPa and 125 MPa) may be caused by the damaged tissue since it can stimulate enzymatic browning reactions [34]. Specifically, air and water thawing required a longer thawing time (Figure 2), so the existed ice crystals may have more time to damage the cell tissue during the thawing process, after which, the compartmentalization of enzymes and substrate was damaged, inducing enzyme browning and eventually reducing the L* value. Further, a significant L* value reduction caused by ultra-high-pressure thawing might be associated with serious tissue damage during the thawing process, which further results in the leakage of cell sap and the enlarged probability of substrate-enzymes contacts [35]. Additionally, ultra-high-pressure treatment may increase the activity of some enzymes, such as latent polyphenol oxidase, or/and released membrane-bound polyphenol oxidase, causing enzymatic browning of thawed mangoes, consequently [36]. In contrast, microwave thawing could prevent color changes due to presumably lower structural damage related to the thawing rate and heating pattern.

Overall, both Δ*E* and L* values of microwave-thawed samples were better than those of mangoes thawed by other approaches and conditions.

### 3.3. Firmness

**Figure 3:**
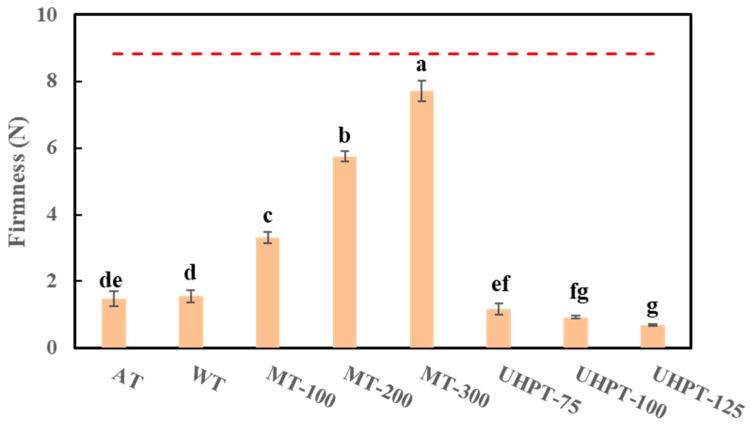
The firmness of thawed mangoes that were affected by different thawing conditions. The dotted line represents the firmness of the fresh mango. The different lowercase letters mean the significant difference among thawing conditions (*p* < 0.05).

Figure 3 reveals that there has been a drop in the firmness of mangoes after the thawing process. This can be partly explained by the ice crystals’ thawing and the related cell tissue damage. Moreover, this result may also be correlated to the activation of cell wall hydrolytic enzymes, since previous research indicated that these enzymes could trigger the degradation of middle lamella as well as the loss of cell adhesion, resulting in the fruits’ softening [37,38].

In addition, Figure 3 shows that the selection of different thawing methods influenced the level of firmness loss, while the microwave-thawed mangoes presented less firmness loss than the others. This result could also be associated with the thawing rate and the cellular damage from ice crystals. Moreover, the firmness of microwave-thawed mango increased significantly with the increase in microwave power level and reached a peak point when 300 W was applied, indicating the 300 W microwave achieved the least firmness loss of thawed mango. However, the pre-experiments observed that when the microwave power level exceeded 300 W, some of the thawed mangoes demonstrated a serious overheating phenomenon (data are not shown). During the thawing process, the frozen mango samples can hardly become homogeneous because they always contained frozen and unfrozen phases. Some components, which differed greatly in their abilities to absorb radiofrequency energy, can hardly be distributed evenly within the samples. This was likely to cause located areas to overheat earlier than other areas had thawed [39]. Similar results were observed in the microwave thawing process of frozen chicken breast and whole rainbow trout [19,40]. When the microwave power approached a certain level, the unbalance of the temperature distribution increased, resulting in more serious overheating of located areas afterward.

Air-thawed and water-thawed mango samples showed lower firmness values and higher firmness loss than microwave-thawed samples. This might be connected to a significantly longer thawing time that allows more cell collapses and enzyme-caused cell wall-degrading [41]. Although ultra-high-pressure thawing had a faster thawing rate, high-pressure-thawed mangoes adversely showed higher firmness loss than the samples thawed by other methods. A higher pressure resulted in a lower firmness value, where 125 MPa high-pressure thawing showed the greatest firmness loss. This result could be explained by the stronger compression force caused by higher pressure, which added to mango tissue and thus caused cell rupture. Early studies found that the texture loss of frozen strawberries could hardly be prevented by applying high-pressure thawing conditions, so the hardness drops were also observed [25].

### 3.4. Drip Loss

During the thawing process, the interior ice crystals of frozen food would stepwise transfer to the water. Certain amounts of water may be absorbed and held by the cells, while the left part of the unabsorbed water would exude from the cells, causing liquid loss [30]. Both water and water-soluble materials that leaked from cells during the thawing process were included in the drip loss [42]. These dripping liquids can provide a good growth medium for bacteria [40]. Therefore, drip loss is considered one of the important indicators which can describe the quality and nutrition preservation of frozen samples after thawing.

**Figure 4:**
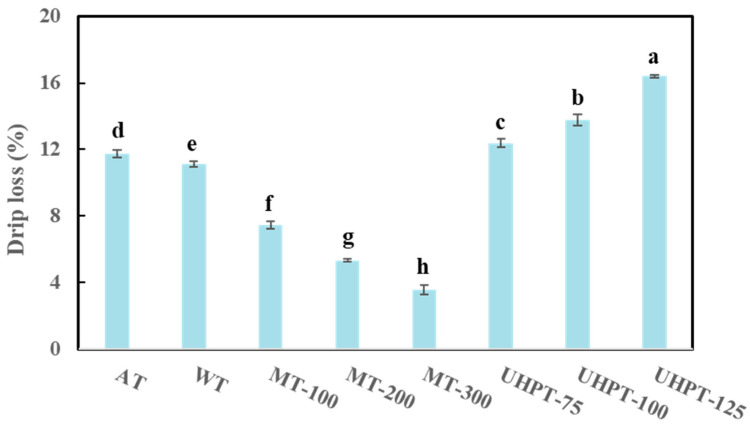
The drip loss of thawed mangoes that were affected by different thawing conditions. The different lowercase letters mean the significant difference among thawing conditions (*p* < 0.05).

As can be seen in Figure 4, the microwave thawing process had significant reductions in drip loss compared with samples thawed by other methods. Moreover, the volume of exuded liquid decreased with increasing microwave power levels. The drip loss of 300 W microwave-thawed samples was the lowest (3.58%) among different power levels. Because of the faster thawing rate of microwave thawing, there was a decrease in drip loss with an increase in the thawing rate. An early study reported that microwave thawing decreased drip loss compared with a slow thawing procedure [19,43]. However, more than a 300 W microwave treatment (data are not shown) resulted in serious drip loss, mainly due to overheating phenomenon. Therefore, this microwave power level should not be used for thawing frozen mangoes. Furthermore, ultra-high-pressure-thawed samples significantly showed the highest drip loss among different thawing methods. The drip loss increased with increasing the pressure, probably due to the cell collapse caused by high pressure. A similar trend of drip loss was also observed once high-pressure thawing was performed with Hami-melon [26]. For the traditional thawing methods, the air and water thawing resulted in a higher drip loss value than microwave thawing, which may be due to the slow thawing process, the decrease in the ability of a cell to hold water, and the increase in drip loss during the long thawing process [41,44]. For water thawing, the drawbacks also included latent microbial contamination and the intensive use of water resources [45]. The change of drip loss is consistent with the results of firmness for mangoes thawed by four different thawing methods.

Overall, a higher drip loss value generally indicates lower product acceptability due to the loss of texture and nutrients of the thawed samples. Accordingly, microwave thawing is the most promising method to reduce the drip loss of frozen mangoes.

### 3.5. Vitamin C Content

**Figure 5:**
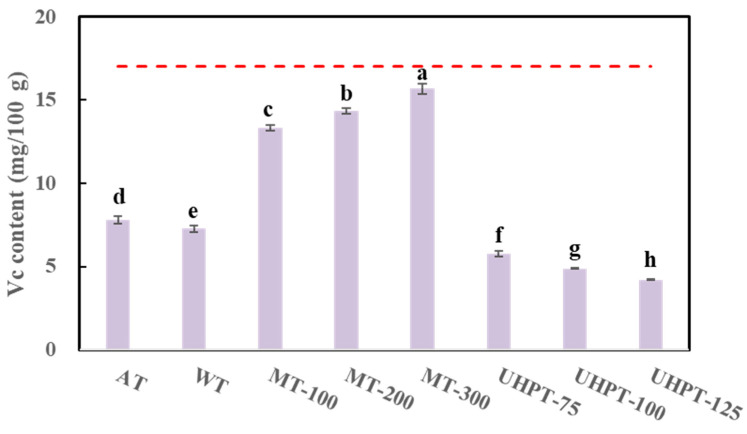
The vitamin C content of thawed mangoes that were affected by different thawing conditions. The dotted line represents the vitamin C content of the fresh mango. The different lowercase letters mean the significant difference among thawing conditions (*p* < 0.05).

Nutritional value is a concealed character that influences consumers’ health in a way that people cannot perceive directly. However, this quality characteristic appears increasingly valued by the consumers [29]. Mango fruits are originally rich in vitamin C. After the freezing and thawing process, the vitamin C content was significantly reduced to different levels, as can be found in Figure 5. The drip loss that occurred during the thawing process of frozen mango may cause not only the loss of water but also the loss of water-soluble materials, including water-soluble vitamin C. Additionally, the enzymatic and non-enzymatic oxidation of ascorbic acid in the presence of oxygen may also cause the reduction [46]. The vitamin C content of mangoes thawed by microwave was significantly higher than that of others. Additionally, vitamin C content increased with the increase in the microwave power level. The 300 W microwave-thawed mango showed the highest vitamin C content among different thawing methods and conditions. This result might be attributed to the faster rate of microwave thawing and shorter exposure to oxygen during thawing under this condition. Similar results were found for faster thawing strawberries of the microwave thawing method [20]. The authors also reported that thawing time is a key factor to improve ascorbic acid retention in samples. In contrast, air and water thawing had lower contents of vitamin C due to the opposite reason. Among all thawing methods, high-pressure thawing had the lowest content of vitamin C, and vitamin C content decreased with increasing pressure. This is probably due to much more drip loss in the samples thawed by the high-pressure thawing method.

### 3.6. Sensory Evaluation

**Figure 6:**
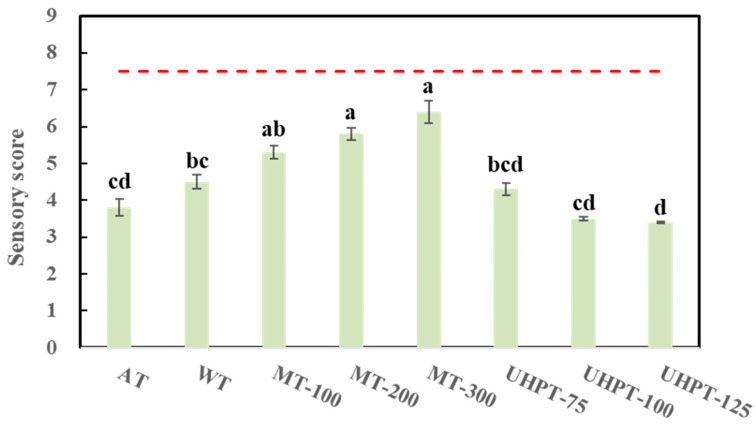
The sensory scores for the overall acceptability of thawed mangoes were affected by different thawing conditions. The dotted line represents the sensory score of the fresh mango. The different lowercase letters mean the significant difference among thawing conditions (*p* < 0.05).

Figure 6 presents the sensory evaluation results on the hedonic scale and the overall acceptability of fresh and all thawed mangoes. The sensory score of fresh mangoes was set as a standard line. The results showed that all the thawed mangoes had reduced scores regardless of the thawing conditions. Moreover, among all the thawing conditions, the results confirmed better acceptability in all microwave-thawed mangoes compared with air-, water- and ultra-high-pressure-thawed mangoes. The sensory score of thawed mangoes reached the highest point (6.4 ± 1.0) among all the samples when 300 W microwave thawing was performed. However, no significant differences were observed between mangoes thawed at 75–100 MPa and those conventionally thawed (air and water thawing).

## 4. Conclusions

Freezing can be a potential solution to minimize the massive loss of mango production if a suitable thawing method is selected to maintain the quality attributes. In this study, microwave thawing (at 100, 200, and 300 W) and ultra-high-pressure thawing (at 75, 100, and 125 MPa), as the representative of thermal and non-thermal thawing methods, were investigated regarding the thawing time and quality attributes of frozen mango, while conventional thawing methods, namely air thawing and water thawing, were compared as the references. The thawing time of the microwave- and ultra-high-pressure thawed samples was significantly reduced as compared to the conventional thawing methods. Ultra-high-pressure thawing showed the least color changes of thawed mangoes, but also the greatest loss of firmness and vitamin C. Based on the comprehensive consideration of all the analyzed quality attributes, the 300 W microwave thawing method achieved the quickest thawing process and the least quality loss regarding the drip, firmness and vitamin C, which is in accordance with the results from the sensory evaluation point of view, and therefore, is considered to be the most favorable condition for thawing mangoes.

## Figures and Tables

**Table 1:** Physicochemical properties of fresh mangoes.

Properties	Mean ± SD
Water content (g/100 g)	86.52 ± 0.24
Total soluble solids (%)	15.80 ± 0.25
Vitamin C content (mg/100 g)	17.04 ± 0.48
Firmness (N)	8.82 ± 0.31
Color parameters	
Lightness (L*)	59.95 ± 0.67
Redness (a*)	17.12 ± 0.29
Yellowness (b*)	82.07± 0.92

**Table 2:** The L*, a*, b* values and total color difference of fresh and thawed mangoes were affected by different thawing conditions.

Thawing Methods	L*	a*	b*	Δ*E*
AT	54.45 ± 1.90 d	18.08 ± 0.51 a	91.19 ± 2.82 ab	11.10 ± 1.23 ab
WT	56.44 ± 0.05 c	16.48 ± 2.01 a	93.65 ± 1.69 a	12.32 ± 1.41 a
MT-100	61.32 ± 0.92 b	14.65 ± 1.06 b	88.52 ± 1.37 bc	7.20 ± 0.90 c
MT-200	65.38 ± 0.56 a	12.90 ± 0.60 c	89.50 ± 1.33 bc	10.12 ± 1.30 b
MT-300	65.91 ± 0.48 a	13.10 ± 0.54 bc	80.24 ± 0.95 d	7.36 ± 0.24 c
UHPT-75	61.09 ± 0.75 b	11.07 ± 0.78 d	77.47 ± 2.00 d	7.63 ± 1.76 c
UHPT-100	55.43 ± 1.00 cd	17.12 ± 0.38 a	87.92 ± 0.60 c	9.64 ± 0.32 b
UHPT-125	52.42 ± 0.37 e	11.91 ± 0.48 cd	92.59 ± 1.52 a	12.67 ± 1.15 a

The different lowercase letters mean the significant difference among thawing conditions (*p* < 0.05).

## Data Availability

The datasets generated for this study are available on request to the corresponding author.
